# Upregulation of M_3_ muscarinic receptor inhibits cardiac hypertrophy induced by angiotensin II

**DOI:** 10.1186/1479-5876-11-209

**Published:** 2013-09-12

**Authors:** Yan Liu, Shu Wang, Chao Wang, Haoxin Song, Hongmei Han, Pengzhou Hang, Yanan Jiang, Lanlan Wei, Rong Huo, Lihua Sun, Xu Gao, Yanjie Lu, Zhimin Du

**Affiliations:** 1 Department of Pharmacology (State-Province key lab of China), Harbin Medical University, Heilongjiang 150081, China; 2 Department of Cardiac Care Unit, The First Affiliated Hospital of Harbin Medical University, Harbin, Heilongjiang 150081, P. R. China; 3 Institute of Clinical Pharmacology of the Second Hospital, Harbin Medical University, Heilongjiang 150081, China; 4 Department of Microbiology, Harbin, Heilongjiang, 150081 P. R. China; 5 Department of Biochemistry, Harbin Medical University, Harbin, Heilongjiang 150081, P. R. China

**Keywords:** Cardiac hypertrophy, M_3_ muscarinic acetylcholine receptor, Angiotensin II, Choline

## Abstract

**Background:**

M_3_ muscarinic acetylcholine receptor (M_3_-mAChR) is stably expressed in the myocardium, but its pathophysiological role remains largely undefined. This study aimed to investigate the role of M_3_-mAChR in cardiac hypertrophy induced by angiotensin II (Ang II) and elucidate the underlying mechanisms.

**Methods:**

Cardiac-specific M_3_-mAChR overexpression transgenic (TG) mice and rat H9c2 cardiomyoblasts with ectopic expression of M_3_-mAChR were established. Models of cardiac hypertrophy were induced by transverse aortic constriction (TAC) or Ang II infusion in the mice *in vivo*, and by isoproterenol (ISO) or Ang II treatment of H9c2 cells *in vitro*. Cardiac hypertrophy was evaluated by electrocardiography (ECG) measurement, hemodynamic measurement and histological analysis. mRNA and protein expression were detected by real-time RT-PCR and Western blot analysis.

**Results:**

M_3_-mAChR was upregulated in hypertrophic heart, while M_2_-mAChR expression did not change significantly. M_3_-mAChR overexpression significantly attenuated the increased expression of atrial natriuretic peptide and β-myosin heavy chain induced by Ang II both *in vivo* and *in vitro*. In addition, M_3_-mAChR overexpression downregulated AT_1_ receptor expression and inhibited the activation of MAPK signaling in the heart.

**Conclusion:**

The upregulation of M_3_-mAChR during myocardial hypertrophy could relieve the hypertrophic response provoked by Ang II, and the mechanism may involve the inhibition of MAPK signaling through the downregulation of AT_1_ receptor.

## Background

Heart failure is a significant cause of mortality and morbidity [1,2]. Cardiac hypertrophy is a common precursor to many forms of heart failure. Despite recent advances in the understanding of the pathogenesis of cardiac hypertrophy, currently available medications could not effectively reverse pathologic cardiac hypertrophy. Therefore, the identification of novel therapeutic targets for cardiac hypertrophy is pivotal for the development of effective treatment strategies. Sympathetic and parasympathetic divisions of the autonomic nervous systems play a major role in the regulation of cardiac function. Thus, increased attention has been paid to the changes in the cardiac autonomic innervation in cardiac hypertrophy and failure [3-5].

Previous studies have shown that the M_3_ muscarinic acetylcholine receptor (M_3_-mAChR) is expressed in the hearts of various species, including human, canine, guinea-pig and rabbit [6-8]. M_3_-mAChR plays important role in the regulation and maintenance of cardiac function and heart disease [9-11]. The cardiac M_3_-mAChR has demonstrated negative chronotropic and inotropic effects [12]. Heart rate (HR) correlates with myocardial oxygen consumption and coronary blood flow in normal and pathologic hearts. High HR is an independent predictor of total and cardiovascular mortality in patients with coronary artery disease and left ventricular systolic dysfunction [13,14]. Furthermore, Lamping *et al.* demonstrated that endothelium-dependent relaxation to acetylcholine in coronary circulation was mediated predominantly by the activation of M_3_-mAChR [15].

Interestingly, the changes in the expression of M_2_-mAChR and M_3_-mAChR were associated with atrial dilation [16]. However, the involvement of M_3_-mAChR in ventricular hypertrophy remains largely unexplored. Therefore, we hypothesized that cardiac M_3_-mAChR has potential effects on cardiac hypertrophy, in addition to improving coronary circulation and reducing HR. To test our hypothesis, we generated cardiac-specific overexpression M_3_-mAChR transgenic (TG) mice and rat H9c2 cell line that overexpressed M_3_-mAChR (TG-H9c2). Using these models, we demonstrated that the upregulation of M_3_-mAChR inhibited cardiac hypertrophy induced by angiotensin II (Ang II).

## Materials and methods

### Animals

The animals were kept under standard animal room conditions (temperature 21 ± 1°C; humidity 55 ± 5%) with food and water unlimited. All experimental protocols were approved by the Experimental Animal Ethic Committee of Harbin Medical University, China. Use of animals followed the Guide for the Care and Use of Laboratory Animals published by the US National Institutes of Health (NIH Publication No. 85–23, revised 1996).

### Construction of M_3_-mAChR overexpression TG mice model

TG mice were generated by using a construct in which the α-myosin heavy chain (α-MHC) promoter drove the exclusive expression of M_3_-mAChR in cardiomyocytes [17]. TG mice and their wild-type (WT) littermates of either sex, 8–12 weeks old, with a body weight of 20–25 g, were used. Age-matched WT and TG mice were randomly divided into 4 groups: (1) vehicle-infused WT mice (WT-CTRL); (2) Ang II-infused WT mice (WT-Ang II); (3) vehicle-infused TG mice (TG-CTRL); (4) Ang II-infused TG mice (TG-Ang II). Ang II (0.6 mg/kg per day) or normal saline (NS) was injected subcutaneously for 14 days as described previously [18].

### Echocardiographic analysis

Mice were anesthetized by intraperitoneal injection of sodium pentobarbital (65 mg/kg) (Sigma, St Louis, MO, USA). Then, transthoracic echocardiography was performed with an echocardiographic system equipped with a 10.0-MHz phase-array transducer (GE Vivid 7, GE, USA). Left ventricular diameter and wall thickness were measured using M-mode tracings as described previously [19].

### Hemodynamic measurements, heart weight (HW) measurement and histological analysis

After treatment with Ang II, mice were anaesthetized by intraperitoneal injection of sodium pentobarbital (65 mg/kg), and then put on a heated platform for body temperature and electrocardiography (ECG) measurements. Pressure-volume control unit FV896B PV catheter (Scisense advancing micro-sensor technology, London, Ontario, Canada) was inserted through the right carotid artery into the aorta for mean arterial blood pressure (MAP) measurement [18]. Next, the hearts were quickly excised and washed with cold (4°C) PBS buffer. The ratio of whole heart weight to body weight (HW/BW) and left ventricular weight to body weight (LVW/BW) was measured. Ventricle tissue was then equally divided into three parts. One part was fixed with 4% paraformaldehyde and then stained with hematoxylin and eosin (HE). The other two parts and atrium tissues were frozen in liquid nitrogen and stored at -80°C for subsequent analysis.

### Transverse aortic constriction

The pressure-overload cardiac hypertrophy model was induced by transverse aortic constriction (TAC) as described previously [20]. Adult mice (BDF1, 24 ± 2 g), were anesthetized by intraperitoneal injection of sodium pentobarbital (65 mg/kg). After successful endotracheal intubation, the cannula was connected to a volume cycled rodent ventilator (UGO BASILE S.R.L. Italy).The transverse aorta was isolated from annexed tissue, and the artery was partially ligated immediately with 7–0 silk around a 25-gauge blunted needle, which was subsequently removed. Sham operated mice underwent the same procedure, except that the transverse aorta was not partially ligated. The chest was closed and the animals were kept ventilated until the recovery of autonomic breath, and then raised for 14 days.

### Preparation of neonatal rat ventricular myocytes (NRVMs)

NRVMs were isolated from 1-day-old neonatal Wistar rat hearts and differentially plated to remove fibroblasts [21]. Ang II (Sigma, St Louis, MO, USA) or isoproterenol (ISO) (Sigma, St Louis, MO, USA) was added to the culture medium at a final concentration of 0.1 μM or 10 μM for 48 h to induce hypertrophy [18,22]. The cells were then harvested to determine M_3_-mAChR expression during cardiac hypertrophy.

### Construction of M_3_-mAChR overexpression TG-H9c2 cell model

pcDNA3.1 (+) human M_3_-mAChR vector was constructed and transfected into rat H9c2 cardiomyoblasts plated on BioFlex plates. After 48 h, cells were cultured in DMEM supplemented with 10% (v/v) heat-inactivated FBS and 500 μg/mL G418 at 37°C in 5% CO_2_ and 95% air, at a relative humidity of 95%. The cells were split 1 to 3 at sub-confluence (70%). Before each experiment, cells were seeded at a density of 5 × 10^4^ cells/cm^2^. H9c2 cells were cultured in serum-free DMEM for 12 h before treatment with or without 0.1 μM Ang II for 48 h. To quantify the cell surface area, the H9c2 cells were stained with acridine orange. The relative surface area of the cells was calculated from the number of pixels by using Image-Pro Plus (version 5.0.1).

### Western blot analysis

Total proteins (~60 μg) were extracted from cells or tissues as described previously [23], fractionated by 10% SDS-polyacrylamide gel electrophoresis and transferred to nitrocellulose membrane. The membrane was incubated at 4°C overnight with the primary antibodies against M_3_-mAChR, M_2_-mAChR (Alomone Labs, Jerusalem, Israel); Ang II type 1 receptor (AT_1_ R) (Sigma, St Louis, MO, USA); c-Jun N-terminal kinases (JNK), phospho-JNK (p-JNK), extracellular regulated protein kinase (ERK), phospho-ERK (p-ERK) and p38 mitogen-activated protein kinase (MAPK) (Santa Cruz Biotechnology, Santa Cruz, CA, USA), phospho-p38 (p-p38) (Cell Signaling Technology, Boston, USA), followed by incubation with the secondary antibody Alexa Fluor 800 rabbit anti-mouse IgG (H + L) or Alexa Fluor 800 goat anti-rabbit IgG (H + L) (Invitrogen, Carlsbad, USA). The images were captured on the Odyssey Infrared Imaging System (LI-COR, Upland, CA, USA) and band intensity (area × OD) was quantified using Odyssey v1.2 software with GAPDH as loading control.

### Real-time quantitative RT-PCR analysis

Total RNA was extracted from the tissues using Trizol reagent (Invitrogen, USA). First-strand cDNA was synthesized by a reverse transcriptase kit (Invitrogen, USA) according to the manufacturer’s instructions, and used as the template for Quatitative RT-PCR analysis on a ABI 7500 fast Real Time system (Applied Biosystems, Foster City, CA, USA), with GAPDH used as an internal control [15]. The primer sequences were as follows: GAPDH, 5′-AAGAAGGTGGTGAAGCAGGC-3′ (forward), 5′-TCCACCACCCTGTTGCTGTA-3′ (reverse); β-myosin heavy chain (β-MHC), 5′-CCAGAAGCCTCGAAATGTC-3′ (forward), 5′-CTTTCTTTGCCTTGCCTTTGC-3′ (reverse); Atrial natriuretic peptide (ANP), 5′-CTCCGATAGATCTGCCCTCTTGAA-3′ (forward), 5′-GGTACCGGAAGCTGTTGCAGCCTA-3′ (reverse); M_3_-mAChR, 5'-CATCATCGGCAACATCCT-3' (forward) and 5'-GAGGTCACAGGCTAAGTTC-3' (reverse).

### Statistical analysis

Data were expressed as means ± SEM and analyzed with SPSS 13.0 software. Comparisons between two groups were made using Student’s t-test. Comparisons among multiple groups were performed using analysis of variance (ANOVA) followed by Bonferroni post test. Differences were considered to be significant at *P* < 0.05.

## Results

### Hypertrophic stimulation increases M_3_-mAChR expression *in vivo* and *in vitro*

**Figure 1:**
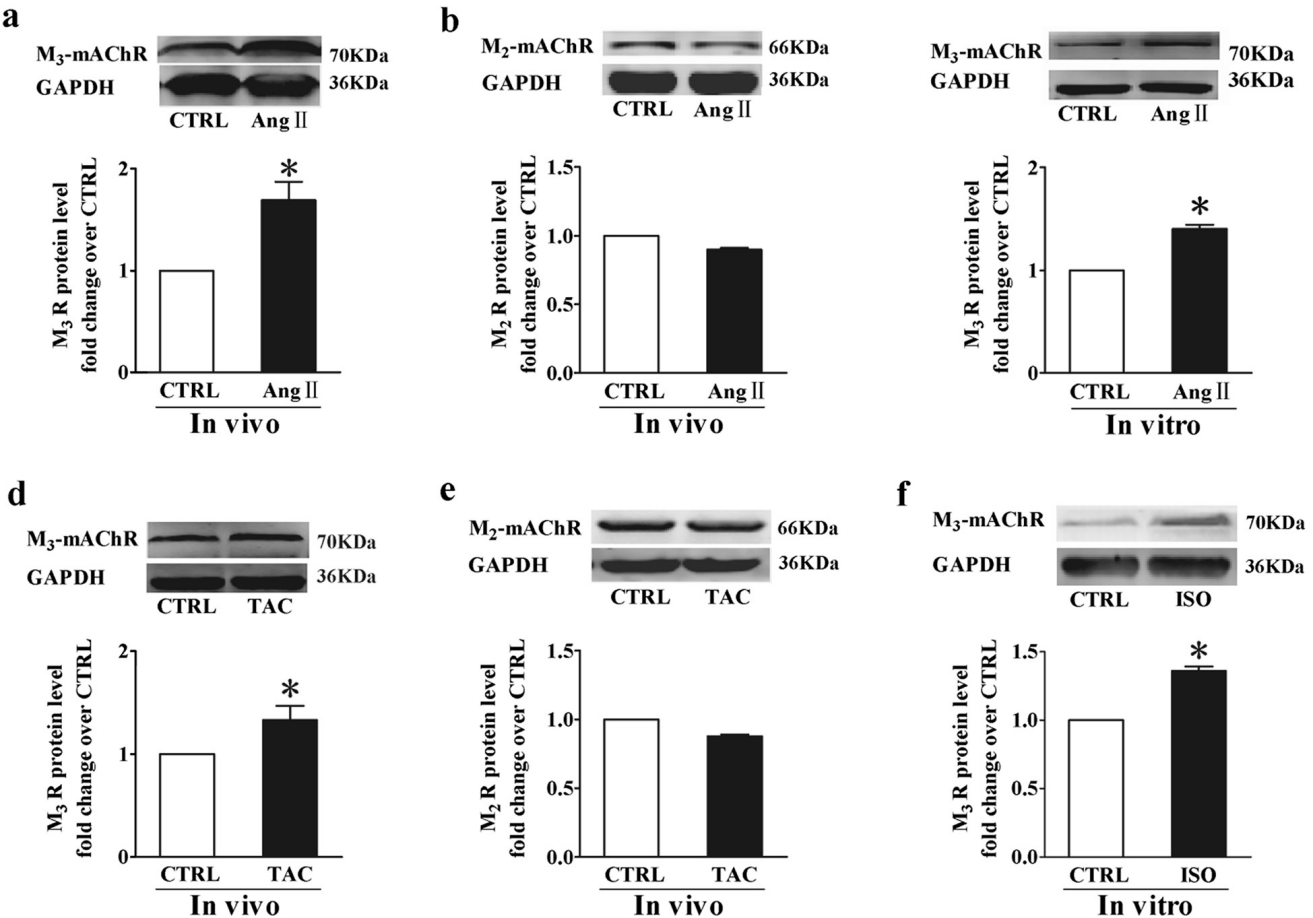
**Upregulation of M**_**3**_**-mAChR expression in *in vitro *and *in vivo *models of hypertrophy. (a**, **b)** Western blot analysis of M_3_-mAChR and M_2_-mAChR protein levels in ventricular tissues from mice with chronic angiotensin II (Ang II) (0.6 mg/kg per day) infusion for 14 days (n = 4 mice per group); **(c)** Western blot analysis of M_3_-mAChR protein level in neonatal rat ventricular myocytes (NRVMs) incubated with Ang II (0.1 μM) for 48 hours (n = 3); **(d**, **e)** Western blot analysis of M_3_-mAChR and M_2_-mAChR protein levels in ventricular tissues from mice with pressure overload by transverse aotic constriction (TAC) for 14 days (n = 4 mice per group); **(f)** M_3_-mAChR protein level from NRVMs incubated with isoproterenol (ISO) (10 μM) for 48 hours (n = 3). GAPDH served as loading control. Values were expressed as mean ± SEM and normalized to the CTRL group. *P < 0.05 vs. CTRL group.

To assess the pathophysiological role of M_3_-mAChR in the heart, we first measured the M_3_-mAChR expression level in the ventricle during cardiac hypertrophy *in vivo*. We observed a 1.69 ± 0.18 fold increase and a 1.33 ± 0.14 fold increase in M_3_-mAChR protein level in the hearts of mice after Ang II infusion or chronic pressure overload via TAC for 14 days, respectively, compared with the control (CTRL) group (Figure 1a and d). However, M_2_-mAChR protein level remained unchanged in both treatment groups (Figure 1b and e). Furthermore, M_3_-mAChR protein level was upregulated after Ang II or isoproterenol (ISO) treatment for 48 h in NRVMs compared with CTRL (Ang II 1.40 ± 0.04; ISO 1.36 ± 0.03) (Figure 1c and f). Besides, RT-PCR analysis confirmed that Chrm3 gene expression was also upregulated in hypertrophic cardiomyocytes (Additional file 1: Figure S1). Taken together, these data suggest that M_3_-mAChR expression is upregulated by hypertrophy both *in vivo* and *in vitro*.

### M_3_-mAChR overexpression exhibits anti-hypertrophic effects in H9c2 cells

**Figure 2:**
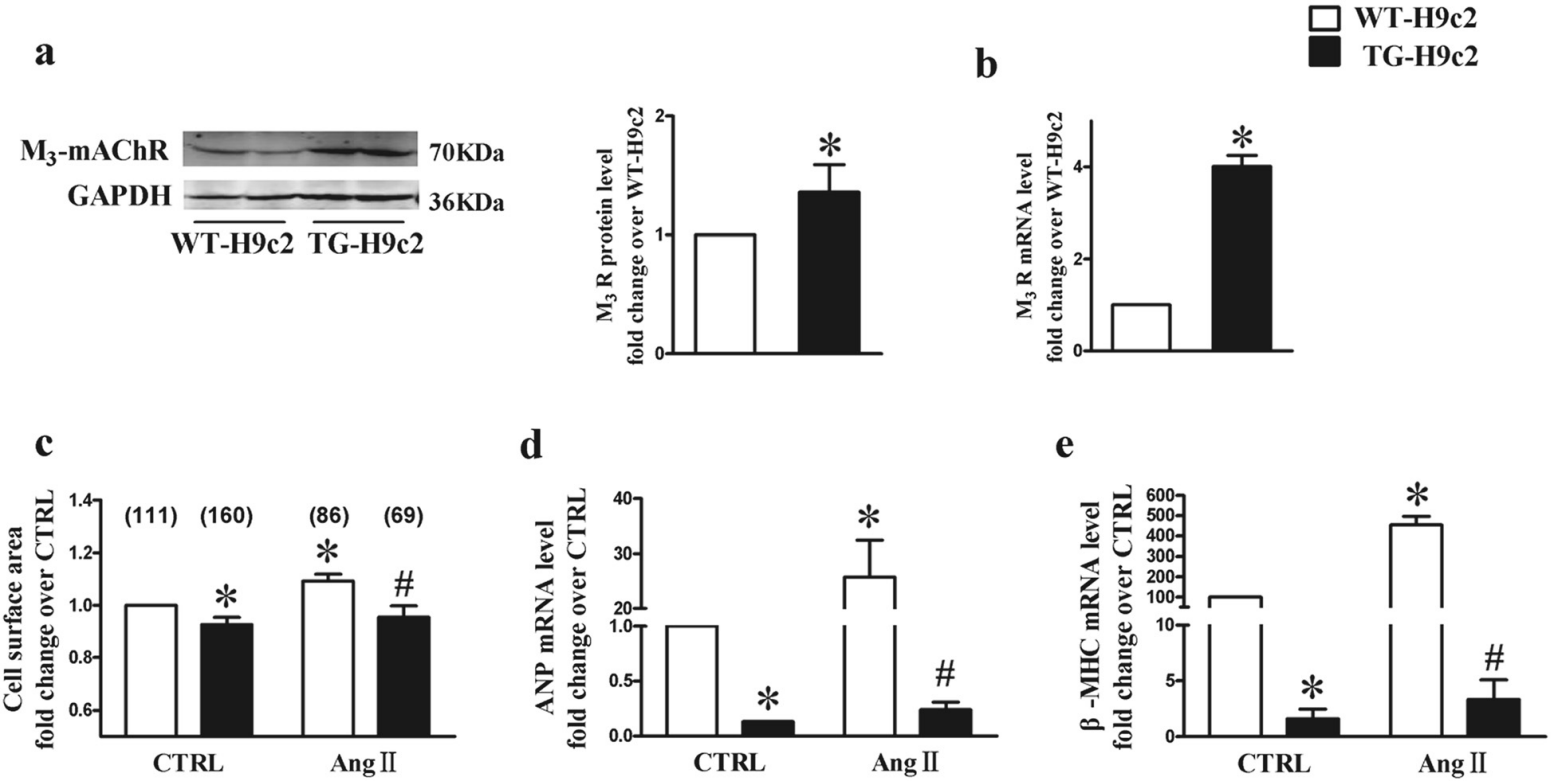
**The effects of M**_**3**_**-mAChR overexpression on Ang II-induced cardiac hypertrophy in H9c2 cells. (a**, **b)** M_3_-mAChR protein and mRNA expression levels were increased in TG-H9c2 cells. TG-H9c2 cell, H9c2 cell line with ectopic expression of M_3_-mAChR; WT-H9c2 cell, wide type H9c2 cell line. Values were expressed as means ± SEM (n = 3) and normalized to the WT-H9c2 group. *P <0.05 vs. WT-H9c2 group. **(c)** M_3_-mAChR overexpression inhibited the increase of cell surface area induced by angiotensin II (Ang II). **(d**, **e)** The mRNA expression of atrial natriuretic peptide (ANP) and β-myosin heavy chain (β-MHC) in TG-H9c2 cells. Values were expressed as means ± SEM (n = 3) and normalized to the WT-CTRL group, *P <0.05 vs. WT-CTRL group; ^#^*P* < 0.05 vs. WT-Ang II group.

To explore the biological significance of M_3_-mAChR expression upregulation in the heart, we compared TG-H9c2 cells with WT-H9c2 cells (Figure 2a and b). The cell surface area was significantly increased after Ang II infusion in WT-H9c2 cells compared with TG-H9c2 cells (WT-Ang II 1.09 ± 0.03 vs. TG-Ang II 0.95 ± 0.04) (Figure 2c). In addition, the mRNA expression levels of ANP and β-MHC, which are cardiac hypertrophy-related genes, were increased by 25.76 ± 6.65 and 4.53 ± 0.43 fold, respectively, after the incubation with Ang II in WT-H9c2 cells but not in TG-H9c2 cells (Figure 2d and e).

### M_3_-mAChR overexpression attenuates cardiac hypertrophy induced by chronic Ang II infusion in mice

**Figure 3:**
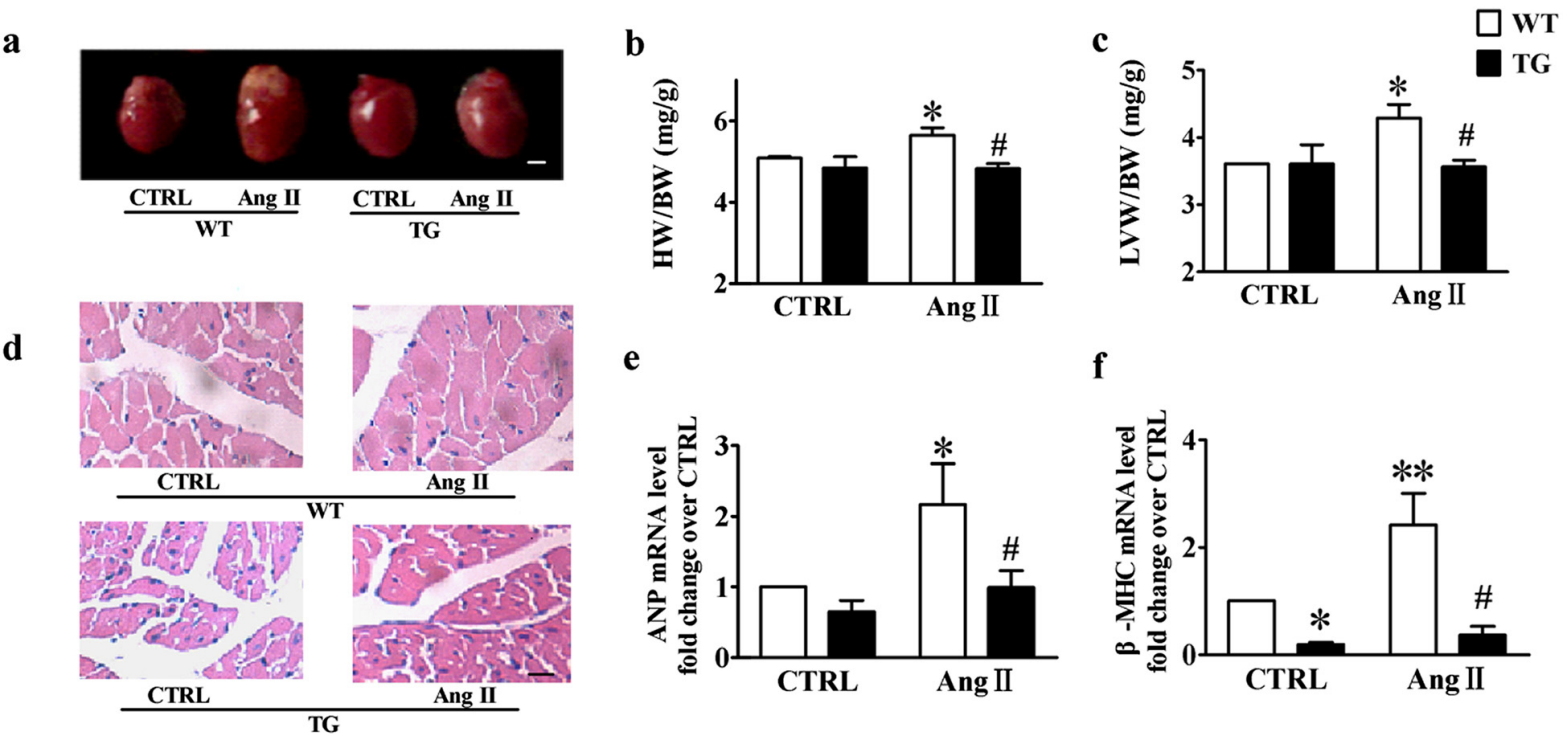
**The effects of M**_**3**_**-mAChR overexpression on Ang II-induced cardiac hypertrophy in cardiac-specific M**_**3**_**-mAChR overexpression mice. (a)** Representative hearts from WT and M_3_-mAChR overexpression transgenic (TG) mice treated with vehicle or angiotensin II (Ang II) (0.6 mg/kg per day) for 14 days. **(b**, **c)** The ratio of heart weight (HW) and left ventricle weight (LVW) to body weight (BW). **(d)** Hematoxylin-Eosin (HE) stained sections of representative hearts. **(e**, **f)**The mRNA expression of atrial natriuretic peptide (ANP) and β-myosin heavy chain (β-MHC). Values were expressed as means ± SEM (n = 6) and normalized to the WT-CTRL group, *P < 0.05 vs. WT-CTRL group; **P < 0.01 vs. WT-CTRL group; ^#^*P* < 0.05 vs. WT-Ang II group. WT-CTRL, vehicle-infused wild type (WT) mice; WT-Ang II, Ang II-infused WT mice; TG-CTRL, vehicle-infused transgenic (TG) mice; TG-Ang II, Ang II-infused TG mice.

To further demonstrate the anti-hypertrophic effects of cardiac M_3_-mAChR *in vivo*, TG mice were generated with cardiac-specific overexpression of M_3_-mAChR. As expected, Ang II infusion induced significant increases in the diastolic intraventricular septum (IVSd), systolic intraventricular septum (IVSs) and diastolic left ventricular posterior wall (LVPWd) thickness of the hearts estimated by echocardiography in WT mice. Meanwhile, HW/BW and LVW/BW ratio, heart rate, and mean arterial pressure (MAP) were also increased (Table 1, Figure 3b and c). However, the cardiac hypertrophic responses to Ang II infusion were completely suppressed in TG mice (Figure 3b and c). HE staining showed that M_3_-mAChR overexpression inhibited Ang II induced cardiomyocyte cross sectional area increase (Figure 3d, Additional file 2: Figure S2). Furthermore, real time RT-PCR analysis showed that the mRNA levels of ANP and β-MHC were increased after Ang II infusion in WT mice compared to those in TG mice (ANP, WT-Ang II 2.17 ± 0.58 vs. TG-Ang II 0.99 ± 0.24; β-MHC, WT- Ang II 2.42 ± 0.59 vs. TG- Ang II 0.36 ± 0.16) (Figure 3e and f). These results indicate that the cardiac hypertrophic effects induced by Ang II infusion were suppressed in TG mice with cardiac-specific overexpression of M_3_-mAChR.

**Table 1:** Bodyweight (BW), heart rate (HR), mean arterial pressure (MAP), ejection fraction (EF) and echocardiographic measurements after Ang II infusion in the mice

**Variable**	**WT-CTRL (n = 6)**	**WT-Ang II (n = 6)**	**TG-CTRL (n = 6)**	**TG-Ang II (n = 6)**
**BW, g**	23.18 ± 1.68	20.93 ± 1.54	23.50 ± 1.15	21.30 ± 1.05
**HR, bpm**	471.74 ± 37.90	642.26 ± 45.85^*^	470.07 ± 34.77	539.20 ± 54.83^#^
**MAP, mmHg**	91.54 ± 3.94	106.57 ± 5.29^*^	99.44 ± 4.02	88.71 ± 3.72^#^
**Echocardiography(n = 5)**				
**IVSd, mm**	0.96 ± 0.05	1.26 ± 0.07^*^	0.86 ± 0.05	1.02 ± 0.06^#^
**IVSs, mm**	1.38 ± 0.06	1.70 ± 0.04^*^	1.36 ± 0.07	1.42 ± 0.09^#^
**LVPWd, mm**	0.94 ± 0.02	1.16 ± 0.07^*^	0.94 ± 0.05	0.90 ± 0.04^#^
**LVPWs, mm**	1.30 ± 0.07	1.44 ± 0.08	1.24 ± 0.04	1.12 ± 0.08^#^
**LVDd, cm**	3.24 ± 0.32	3.24 ± 0.16	3.02 ± 0.20	3.00 ± 0.12
**LVDs, cm**	2.00 ± 0.05	1.84 ± 0.04	1.68 ± 0.04	1.72 ± 0.06
**EF**	0.75 ± 0.02	0.80 ± 0.04	0.81 ± 0.02	0.77 ± 0.08

*IVSd* diastolic intraventricular septum thickness, *IVSs* systolic intraventricular septum thickness, *LVPWd* diastolic left ventricular posterior wall thickness, *LVPWs* systolic left ventricular posterior wall thickness, *LVDd* diastolic systolic left ventricular diameter, *LVDs* systolic left ventricular diameter, *EF* ejection fraction. Values were expressed as means ± SEM (n = 6), ^*^P < 0.05 vs. WT-CTRL group; ^#^*P* < 0.05 vs. WT-Ang II group. WT-CTRL, vehicle-infused wild type (WT) mice; WT-Ang II, angiotensin II (Ang II)-infused WT mice; TG-CTRL, vehicle-infused transgenic (TG) mice; TG-Ang II, Ang II-infused TG mice.

### M_3_-mAChR downregulates AT_1_ R expression and the activation of MAPK signaling in TG mice

**Figure 4:**
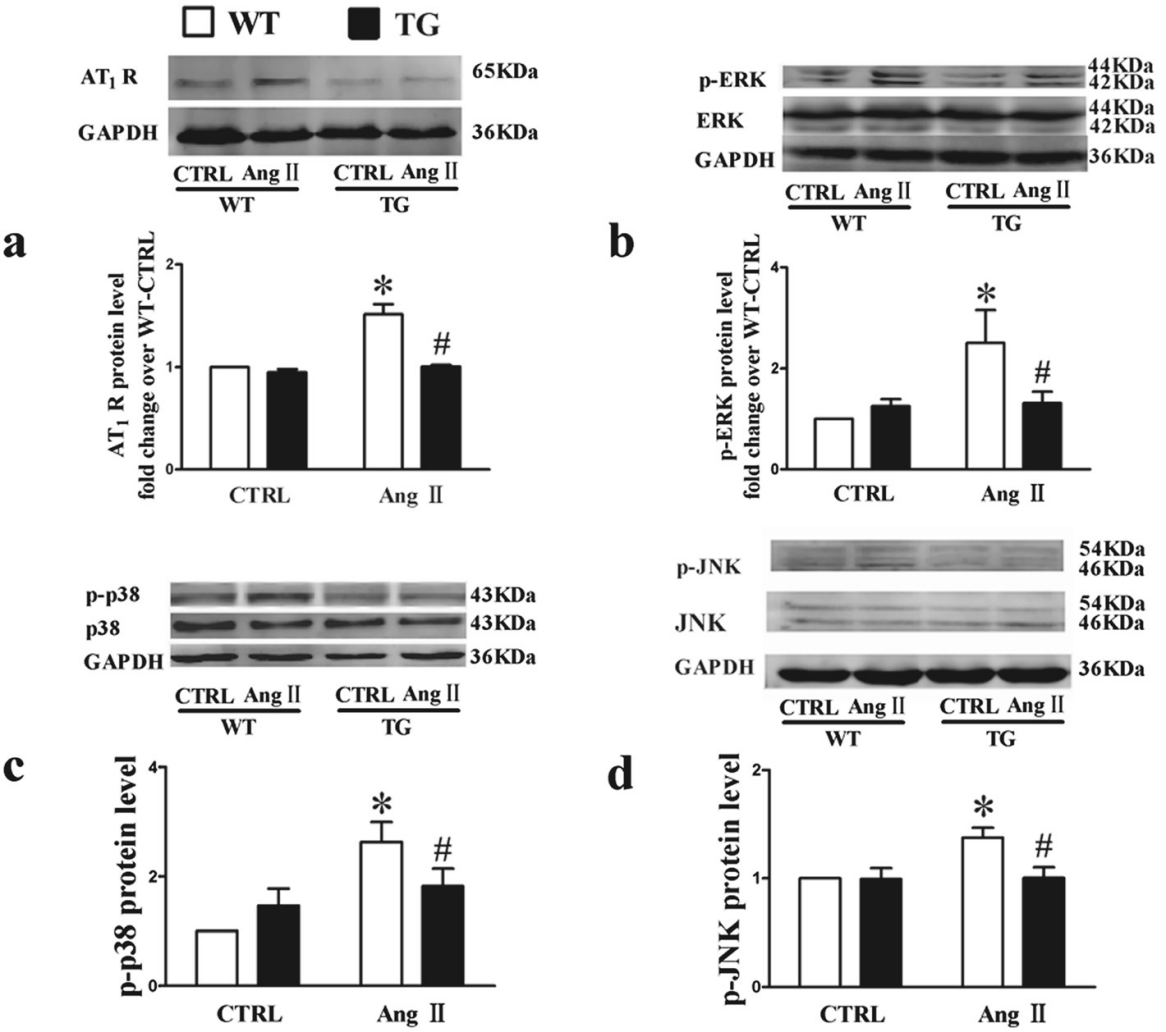
**The changes of AT**_**1 **_**R, p-ERK1/2, p- p38-MAPK, and p-JNK levels in cardiac-specific M**_**3**_**-mAChR overexpression mice. (a)** Western blot analysis of angiotensin II type 1 receptor (AT_1_ R) protein level in WT and TG mice treated with vehicle or Ang II (0.6 mg/kg per day) for 14 days. The increased phospho-extracellular regulated protein kinase (p-ERK) **(b)**, phospho-p-p38 (p-p38) level **(c)** and phospho- c-Jun N-terminal kinases (p-JNK) level **(d)** induced by angiotensin II (Ang II) infusion were attenuated in TG mice. Phospho-protein levels were relative to total protein levels, while total protein levels were relative to GAPDH. GAPDH served as loading control. Values were expressed as mean ± SEM (n = 6) and normalized to the CTRL group. Values were expressed as means ± SEM. *P < 0.05 vs. WT-CTRL group; ^#^*P* < 0.05 vs. WT-Ang II group. WT-CTRL, vehicle-infused wild type (WT) mice; WT-Ang II, Ang II -infused WT mice; TG-CTRL, vehicle-infused transgenic (TG) mice; TG-Ang II, Ang II-infused TG mice.

To determine the role of AT_1_ R in the anti-hypertrophic effects mediated by M_3_-mAChR upregulation, we measured AT_1_ R protein expression level in mice. Western blot analysis showed that Ang II induced the upregulation of AT_1_ R protein expression compared with CTRL in WT mice (Figure 4a). However, Ang II failed to induce the upregulation of AT_1_ R protein expression in TG mice with overexpression of M_3_-mAChR. As the downstream effector of AT_1_ R signaling pathway, MAPK plays an important role in the development of cardiac hypertrophy [24-26]. To further explore the underlying molecular mechanism, the basal and activated levels of JNK, ERK and p38 MAPK were examined in transgenic hypertrophic mice. The levels of p-JNK, p-ERK and p-p38 MAPK were increased in WT mice after Ang II treatment (Figure 4b to d), indicating the activation of MAPK signaling. However, Ang II induced activation of MAPK signaling was abolished in TG mice because the levels of p-JNK, p-ERK and p-p38 MAPK were not significantly increased in these mice.

## Discussion

In the present study we reported several interesting findings: (1) M_3_-mAChR is upregulated during Ang II-induced cardiac hypertrophy both *in vivo* and *in vitro*; (2) Upregulation of M_3_-mAChR could inhibit cardiac hypertrophy induced by chronic Ang II infusion; (3) Upregulation of M_3_-mAChR is mediated by decreased phosphorylation of ERK, JNK, and p38 MAPK cascades through the downregulation of AT_1_ R. These results provide novel insight into the mechanisms underlying the cardioprotective effects of M_3_-mAChR in cardiac hypertrophy, and indicate that M_3_-mAChR is a potential therapeutic target.

Previous studies have demonstrated that the expression of M_3_-mAChR is upregulated during ventricular myocardial ischemia and atrial fibrillation [10,11,27]. Similarly, in the present study, we found that the protein level of M_3_-mAChR was upregulated during cardiac hypertrophy both *in vivo* and *in vitro*. As a control, we detected M_2_-mAChR protein expression and found no significant change in hypertrophic heart. These data indicate the specific role of M_3_-mAChR in cardiac hypertrophy.

The activation of AT_1_ R through the renin-angiotensin system (RAS) plays an important role in the development of cardiac hypertrophy [28,29]. Ang II not only regulates the vasculature, but also promotes the growth of cardiac tissues, resulting in myocardial hypertrophy independently of hypertension [26]. The antagonist of AT_1_ R is widely used for the treatment of hypertension, and is an important drug for the prevention of cardiac remodeling after myocardial infarction. Ang II activates several intracellular signaling pathways such as MAPK signaling [25]. The p38 MAPK cascade has been shown to play a critical role in the pathogenesis of cardiac hypertrophy [25,26,29]. Liu *et al*. have demonstrated that acetylcholine prevented Ang II-induced apoptosis of H9c2 cells via the downregulation of AT_1_ R, the inhibition of ROS-mediated p38 MAPK activation, as well as the regulation of Bcl-2, Bax and caspase-3 [24]. In addition, previous study showed that M_3_-mAChR was closely related to ERK and p38 MAPK [30]. In the present study we found that the phosphorylation levels of ERK1/2, JNK and p38 were increased in response to hypertrophic stimulation in WT mice but not in TG mice, consistent with previous studies that the p38 MAPK and ERK1/2 were involved in cardiac hypertrophy [2]. Taken together, these data suggest that MAPK signaling is crucially involved in the regulation of cardiac myocytes by M_3_-mAChR. Suppression of the MAPK signaling by M_3_-mAChR through the downregulation of AT_1_ R in the heart may attenuate cardiac remodeling.

It is worth mentioning that the physiological parameters, such as BW, HR, cardiac functions, and HW/BW and LVW/BW ratio, are comparable at baseline in WT and TG mice, thus the effects of cardiac-specific overexpression of M_3_-mAChR are likely to be exerted only in the context of hypertrophy stimulation.

In summary, the current study provides new insight into the role of M_3_-mAChR upregulation in the development of cardiac hypertrophy induced by Ang II. Our findings suggest that M_3_-mAChR functions as an endogenous negative regulator of hypertrophic response, thus representing a novel therapeutic target for cardiac hypertrophy. Additional studies to elucidate the molecular mechanisms of the anti-hypertrophic properties of cardiac M_3_-mAChR will promote the application of M_3_-mAChR regulators in the clinic.

## Competing interests

The authors declare they have no competing interests of this article.

## Authors’ contributions

YL participated in the design of the study, performed the experiments in vivo and drafted the manuscript. SW, CW, HS participated in the experiments *in vitro*. HH PH performed the statistical analysis. YJ, LW, RH, LS, XG performed real-time PCR assay. ZD conceived of the study, helped to draft the manuscript and provided supervision. YL conceived of the study, participated in its design and provided supervision. All authors read and approved the final manuscript.

## Supplementary Material

Additional file 1: Figure S1 Upregulation of M_3_-mAChR mRNA expression in *in vitro* and *in vivo* models of hypertrophy. (a) M_3_-mAChR mRNA levels in ventricular tissues from mice with chronic angiotensin II (Ang II) (0.6 mg/kg per day) infusion for 14 days (n = 4 mice per group); (b) M_3_-mAChR mRNA level from neonatal rat ventricular myocytes (NRVMs) incubated with isoproterenol (ISO) (10 μM) for 48 hours (n = 4). Values were expressed as mean ± SEM and normalized to the CTRL group. *P < 0.05 vs. CTRL group. Click here for file

Additional file 2: Figure S2 M_3_-mAChR overexpressioninhibited angiotensin II (Ang II) induced cardiomyocyte cross-sectional area increase. Values were expressed as means ± SEM and normalized to the WT-CTRL group, *P < 0.05 vs. WT-CTRL group. ^#^P < 0.05 vs. WT-Ang II group. WT-CTRL, vehicle-infused wild type (WT) mice; WT-Ang II, Ang II-infused WT mice; TG-CTRL, vehicle-infused transgenic (TG) mice; TG-Ang II, Ang II-infused TG mice. Click here for file
