# Supplementary figures and images for: Upregulation of M3 muscarinic receptor inhibits cardiac hypertrophy induced by angiotensin II
Source: J Transl Med. 2013 Sep 12;11:209. doi: 10.1186/1479-5876-11-209 (PMC3819674; doi:10.1186/1479-5876-11-209)

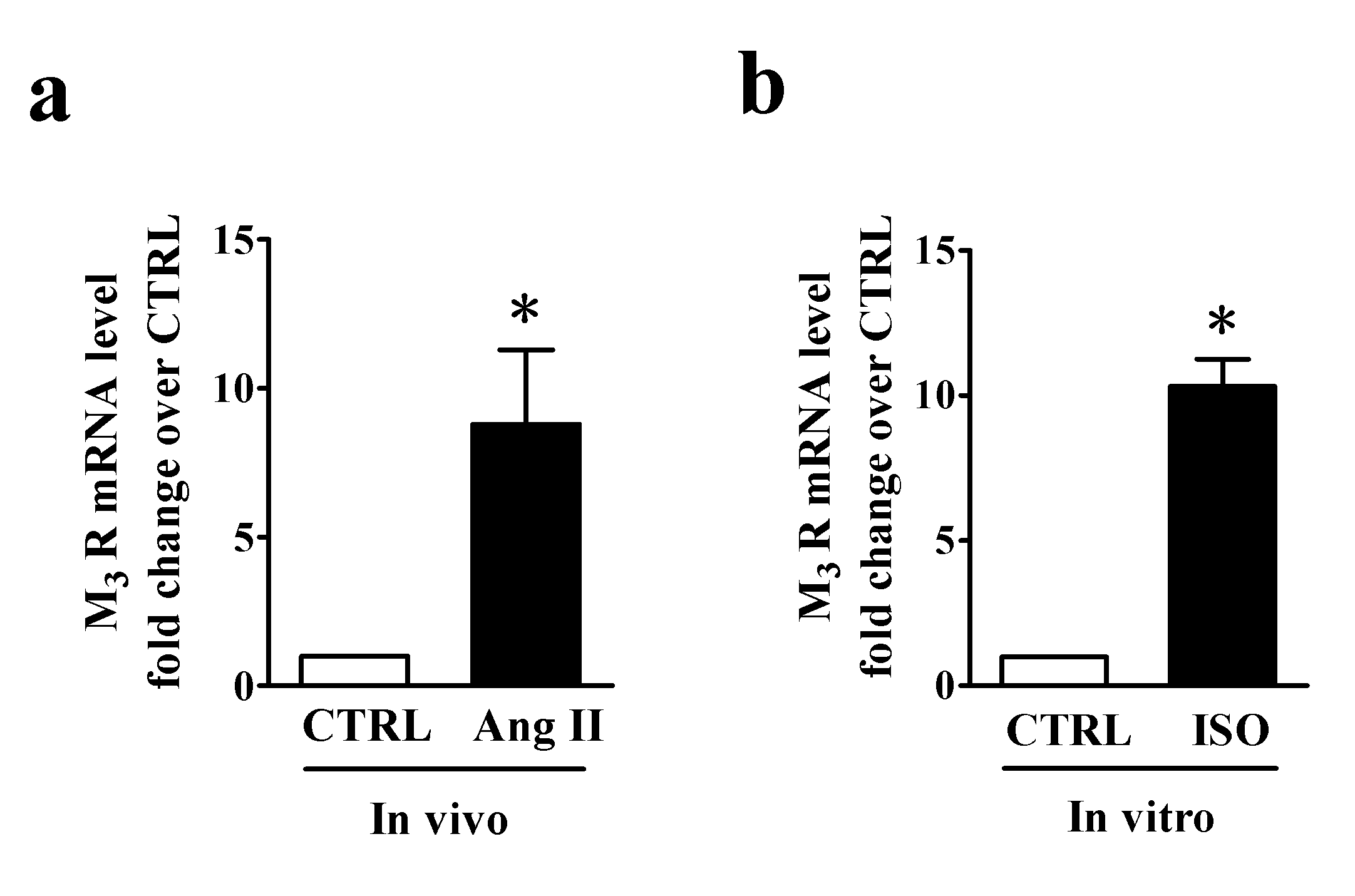

Supplement: Additional file 1: Figure S1 — Upregulation of M3-mAChR mRNA expression in in vitro and in vivo models of hypertrophy. (a) M3-mAChR mRNA levels in ventricular tissues from mice with chronic angiotensin II (Ang II) (0.6 mg/kg per day) infusion for 14 days (n = 4 mice per group); (b) M3-mAChR mRNA level from neonatal rat ventricular myocytes (NRVMs) incubated with isoproterenol (ISO) (10 μM) for 48 hours (n = 4). Values were expressed as mean ± SEM and normalized to the CTRL group. *P < 0.05 vs. CTRL group. [file 1479-5876-11-209-S1.tiff]

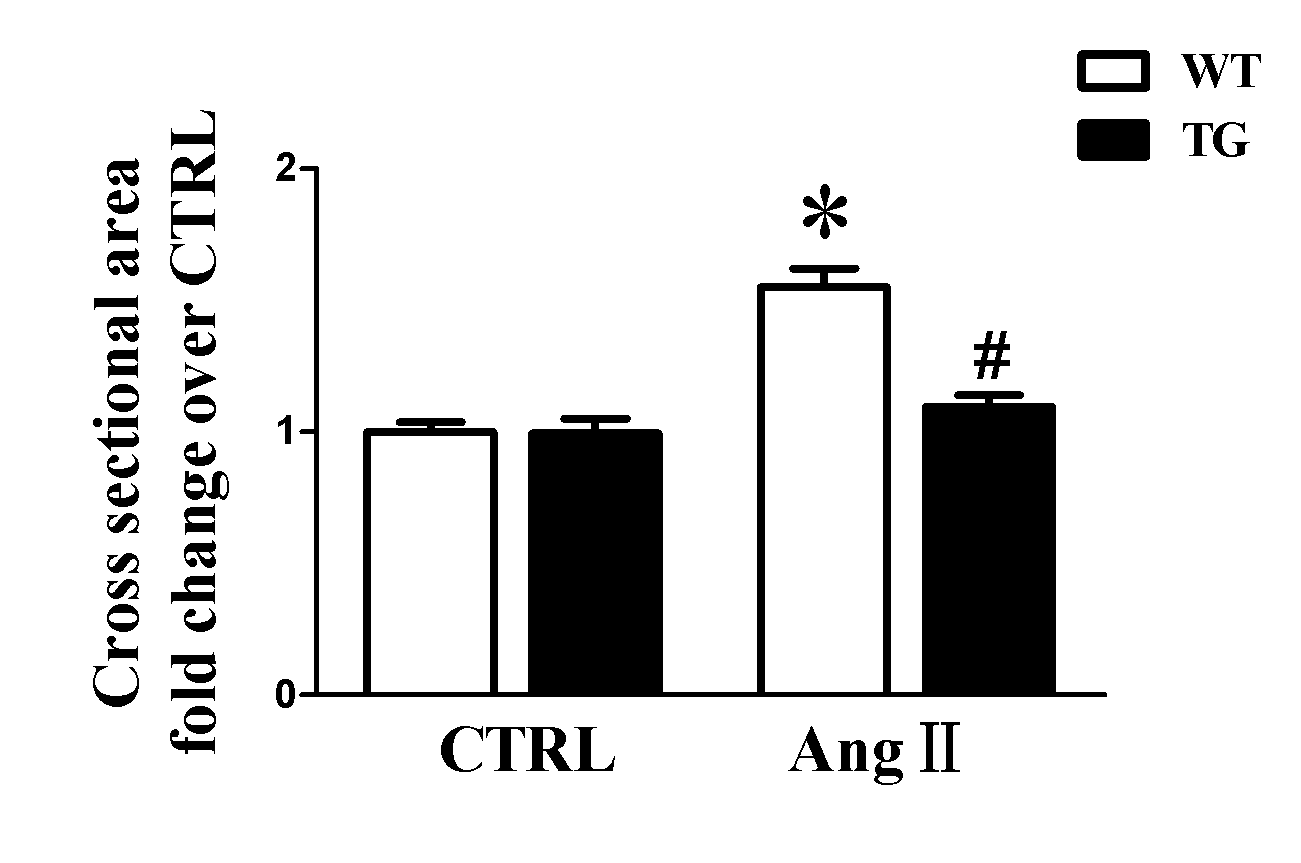

Supplement: Additional file 2: Figure S2 — M3-mAChR overexpressioninhibited angiotensin II (Ang II) induced cardiomyocyte cross-sectional area increase. Values were expressed as means ± SEM and normalized to the WT-CTRL group, *P < 0.05 vs. WT-CTRL group. #P < 0.05 vs. WT-Ang II group. WT-CTRL, vehicle-infused wild type (WT) mice; WT-Ang II, Ang II-infused WT mice; TG-CTRL, vehicle-infused transgenic (TG) mice; TG-Ang II, Ang II-infused TG mice. [file 1479-5876-11-209-S2.tiff]
